# ﻿A new white-flowered species of *Gagea* (Liliaceae) from the Fergana Valley, Uzbekistan and Kyrgyzstan

**DOI:** 10.3897/phytokeys.260.151373

**Published:** 2025-07-28

**Authors:** Orzimat Turdimatovich Turginov, Igor Germanovich Levichev, Gulsauir Tanirbergen qizi Kurbaniyazova, Farhod Isomiddinovich Karimov, Lei Yang, Wen Jun Li

**Affiliations:** 1 Institute of Botany, Academy of Sciences Republic of Uzbekistan, Tashkent, 100125 str., Durmon yuli 32, Uzbekistan Institute of Botany, Academy of Sciences Republic of Uzbekistan Tashkent Uzbekistan; 2 State Key Laboratory of Ecological Safety and Sustainable Development in Arid Lands, Xinjiang Institute of Ecology and Geography, Chinese Academy of Sciences, No.818 South Beijing Road, Urumqi 830011, China Xinjiang Institute of Ecology and Geography, Chinese Academy of Sciences Urumqi China; 3 V.L. Komarov Botanical Institute of the Russian Academy of Sciences BIN RAS 197376 St. Petersburg, str., prof. Popov, 2, Russia V.L. Komarov Botanical Institute of the Russian Academy of Sciences St. Petersburg Russia; 4 China-Tajikistan Belt and Road Joint Laboratory on Biodiversity Conservation and Sustainable Use, Xinjiang Institute of Ecology and Geography, Chinese Academy of Sciences, No.818 South Beijing Road, Urumqi 830011, China Xinjiang Institute of Ecology and Geography, Chinese Academy of Sciences Ürümqi China

**Keywords:** Ageotropic sclerified roots, etymology, Fergana Valley, holotype, new species of *Gagea*, sect. *Incrustata*

## Abstract

The article describes the morphological and anatomical features of *Gageakhassanovii* Levichev, Turginov & W.J.Li, **sp. nov.**, recently described from the Fergana Valley. The species belongs to the section Incrustatae and differs from the species *G.circumplexa* Vved. in the colour of the flower and the presence of hairs on the leaves of the roots. This species is considered endemic to the Fergana Valley and contributes to the region’s floristic uniqueness.

## ﻿Introduction

The Fergana Valley and the Syr-Darya River serve as natural geographical boundaries dividing the floristic regions of the Pamir-Alay and the Tien-Shan mountain systems. This area represents a key centre of species diversity for the genus *Gagea* Salisb., which is widely distributed from the Atlantic to the Pacific Ocean. Based on floristic and taxonomic analyses, two major centres of species richness within *Gagea* have been identified in Central Asia (Levichev 1999a). Of the more than 320 species currently accepted in the genus (Levichev et al. 2019, 2023), 105 are recorded from the Pamir-Alay and 73 from the Western Tien Shan, with 34 taxa shared between the two, predominantly inhabiting the lower montane and submontane belts of valley systems.

**Figure 1.**
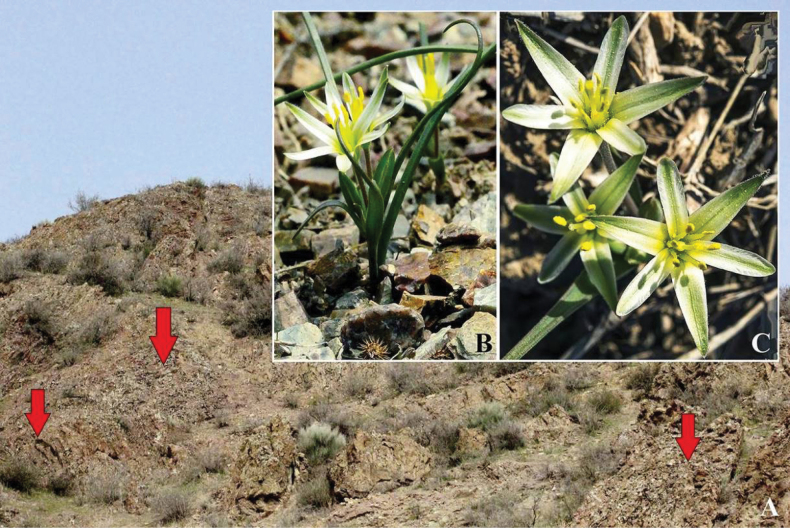
**A.** View of the classic habitat of *G.khassanovii* (arrows indicate the position of individual); **B.***G.khassanovii* from the foothills of the Alay and **C.** Chatkal (Sumsar) ranges. Photo: **A, B.** O.Turginov, C-A. Zhdanko.

**Figure 2.**
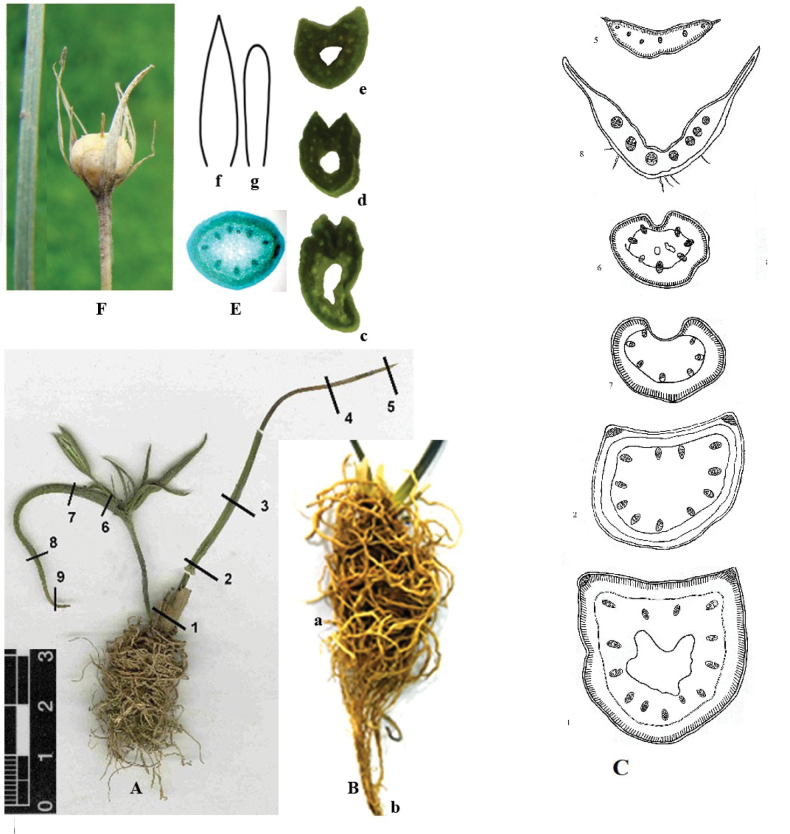
Morphological features of *G.khassanovii*. **A.** General view; **B.** A bulb covered with soil braid of ageotropic sclerified (a) and geotropic feeding with (b) roots; Cross - sections of dry samples; **C.** The basal leaf (1–5); **D.** The lower leaf under the inflorescence (6–9, see the marks in **A**); c, e. sections of the basal leaf (fresh herbarium, c-below the middle, d. in the middle, e-above the middle); **E.** Section of the peduncle; **F.** Mature bud; Tepals: f. outer, g. inner.

Within the Fergana Valley, 56 species of *Gagea* have been documented, including several local endemics exhibiting restricted distributions and distinct morphological characteristics. Of particular interest are *Gageaincrustata* Vved. and *G.circumplexa* Vved., both representatives of sect. Incrustatae Levichev. The former is confined to the Mogoltau ridge and the Kasansai–Kuramin tract, whereas the latter is restricted to the western foothills of the Alay ridge. The present study describes a third narrowly endemic taxon from this section, *Gageakhassanovii* Levichev, Turginov & W.J.Li, sp. nov. (Figs 1, 2), distinguished by its white flowers and morphological affinity to *G.circumplexa*.

## ﻿Material and methods

### ﻿Study area and fieldwork

Field investigations were conducted in March 2019 in the south-western part of the Fergana Valley, specifically in the Imam-ata Range (Alay Mountains, Uzbekistan), at an altitude of 900–1000 m a.s.l. Surveys were carried out using a route method. For each locality, habitat conditions, population size, plant associations, slope and soil type were recorded. Taxonomic literature (Vvedensky and Kovalevskaya 1971) was consulted to support species identification and comparative analysis. The coordinates of each population were recorded using a Garmin eTrex 30x GPS device and spatial mapping was performed using ArcGIS v.10.8 (ESRI, Redlands, CA, USA) to determine distribution patterns and elevation gradients.

### ﻿Morphological and anatomical studies

Morphological analysis was based on herbarium specimens and freshly-collected plants. Diagnostic characters such as bulb structure, stem morphology, leaf shape and size, inflorescence architecture, tepal morphology and flower colour were measured using digital calipers (accuracy ± 0.01 mm). For anatomical study, complete plants were preserved in 70% ethanol at the budding stage. Cross-sections of basal stem, peduncle and leaf were prepared manually using a straight razor, stained with methylene blue and mounted in glycerine. Anatomical structure was examined under a Leica DM1000 microscope following Barykina and Chubatova (2005).

### ﻿Molecular investigation

Leaf tissue was collected and dried in silica gel for DNA extraction. DNA was isolated using the Plant Genomic DNA Kit (DP320, Tiangen Biotech, Beijing). Library preparation was carried out using the Nextera DNA HT Sample Prep Kit (Illumina, USA) according to the manufacturer’s instructions. Sequencing was performed using Illumina HiSeq with paired-end reads (2 × 150 bp). Raw reads were assembled using GetOrganelle (Jin et al. 2020) and SPAdes v.3.10.1 (Bankevich et al. 2012). To improve the quality of multiple sequence alignments, raw reads were trimmed using trimAl (Capella-Gutiérrez et al. 2009). We retrieved four plastid regions (*rbcL*, *matK*, *trnL-F* spacer and *psbA-trnH*) and the nuclear ITS region (Allen et al. 2003; Ronsted et al. 2005; Zarrei et al. 2009; etc.). Reference sequences of related *Gagea* species were downloaded from GenBank (Liu et al. 2012). Bayesian and Maximum Likelihood phylogenies were inferred using IQ-TREE and MrBayes, based on concatenated plastid regions and *ITS*, following Ronquist et al. (2012) and Nguyen et al. (2015). Multiple sequence alignments were performed using MAFFT v.7 (Katoh and Standley 2013; Zhang et al. 2020). The best-fitting nucleotide substitution models were selected using ModelFinder (Kalyaanamoorthy et al. 2017). Phylogenetic trees were constructed using IQ-TREE (Minh et al. 2020) and MrBayes, and visualized with iTOL v.5 (Rambaut 2014; Wick et al. 2015; Letunic and Bork 2021). Sequence data were also managed and analyzed using Geneious Basic v.5.6 (Kearse et al. 2012). The assignment of species to sections in the phylogenetic tree follows the updated sectional classification of *Gagea* proposed by Peterson et al. (2019). Voucher information and GenBank accession numbers are provided in Suppl. materials 1, 2.

## ﻿Results

### ﻿Morphological comparison

Key morphological differences between *Gageakhassanovii* and *G.circumplexa* are summarised in Table 1. Notably, *G.khassanovii* has white tepals, longer basal and cauline leaves relative to its inflorescence and distinct bulb characteristics.

**Table 1.** Comparison of morphological characters between *Gageakhassanovii* and its morphologically similar species.

Characters	*Gageacircumplexa*	*Gageakhassanovii*
Facies habitus	Solitary, **yellow-flowered** plants, mainly on clayey, **fine earths** of plains and foothills.	Solitary, **white-flowered** plants, mainly on **strongly gravelly** soils and **rocks** of mountain spurs.
Bulb	The bulb is tightly braided with thick (sclerified) roots and the membranes are extended into a long, longitudinally split neck.	The bulb is tightly braided with thick (sclerified) roots and the membranous layers are continued into a small, longitudinally split neck.
Radical leaf	The root leaf is one, linear (unifacial), rounded in cross section and flat-concave from above, with a small cavity longer than the inflorescence.	The root leaf is one, linear (unifacial), **elongatedly** rounded in cross section and **deeply grooved** from above, with a **voluminous cavity** that is **two or more times longer** than the inflorescence.
The second root leaf is under the inflorescence.	The second root leaf grows together with the peduncle and separates from it as (in the form of) a subflowering, bifacial, narrow-grooved leaf with a relatively short apical point, approximately equal to or slightly longer than the inflorescence.	The second root leaf grows together with the peduncle and separates from it as (in the form of) a subflowering, bifacial, narrow-grooved leaf with a **long, cylindrical** apical point, **much longer** than the inflorescence.
Inflorescence	The inflorescence is often **multi-flowered.**	The inflorescence is usually **1–3-flowered.**
Perianth	The outer and inner tepals are **lanceolate**, **equally pointed, yellow**, broadly green along the back, slightly shorter inner, wide **yellow-bordered** outside.	Outer and inner tepals of **various shapes**: outer lanceolate, **sharp**; inner **linear, rounded at the end; milky-white**; green on the back; inner broadly **white-bordered**.

### ﻿Molecular phylogenetics

**Figure 3.**
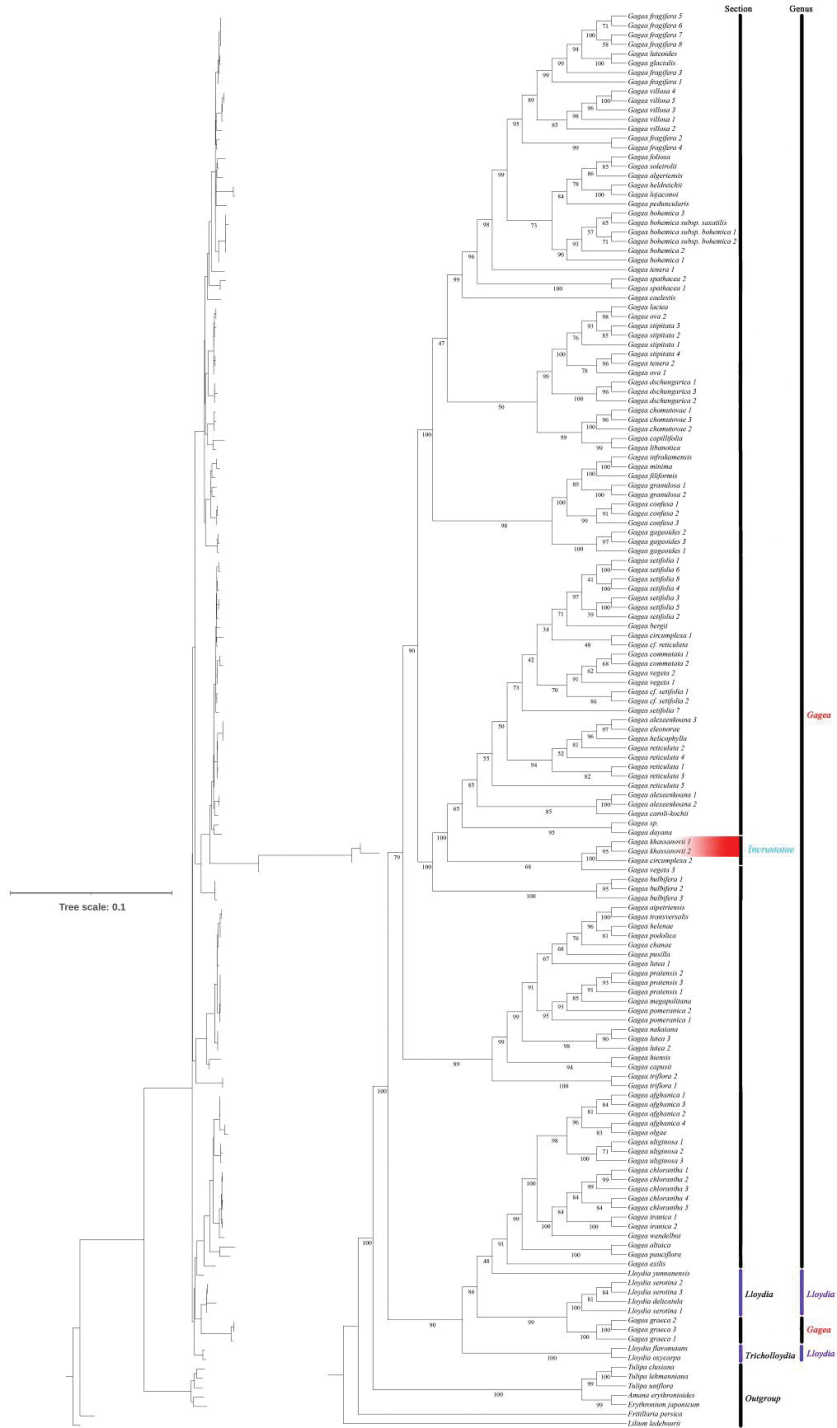
Phylogeny inferred by Maximum Likelihood (ML) and Bayesian Inference (BI). The figure is organised into two main parts: the branch length diagram and the detailed phylogenetic tree. Support values displayed on the branches.

The two individual putative new species are deeply nested within Gageasect.Incrustatae and form a sister group to *G.circumplexa*. ML and BI topologies were also representing high support, all of which had support values of 100 or 0.999 respectively (Fig. 3, Suppl. material 3).

### ﻿Taxonomy treatment

#### ﻿ Gagea khassanovii

Taxon classificationPlantaeLiliales

Levichev, Turginov & W.J.Li sp. nov.

5FC53F9F-E1BC-5265-886B-173728884355

urn:lsid:ipni.org:names:77366291-1

Figs 2 , 4

##### Holotype.

Uzbekistan • Andijan Region. Fergana Valley, Iman-Ata Mountains, Alay ridge (foothills), Rocks; 904 m.a.s.l.; 12 March 2020; 40°53'52.3"N, 72°14'04.9"E; Leg.: *Turginov, Rakhmatov* (TASH: TASH032651!, iso- LE: TASH032652!).

##### Diagnosis.

The species is similar to *G.circumplexa* Vved. in habit, but can be distinguished from the latter by the following characters: perianth is white (vs. golden yellow); inner tepals are linear, rounded at the apex (vs. lanceolate, pointed); cauline and basal leaves are much longer than the inflorescence (vs. equal to or slightly longer) (Table 1).

##### Description.

Single, squat, with white tepals of the plant. The bulb is 9–20 mm in diameter, round, tightly braided with sclerified roots, covered with grey-brown tunics, continued in a filmy neck (10–25 mm long). There is no vegetative reproduction. Peduncle 3–8 cm long, half submerged in the soil, in cross section rounded, about 1 mm in diameter. The basal leaf is single, linear, 2–2.5 times larger than the inflorescence, about 1.5–2 mm wide, on the cross section grooved-semicircular with a large cavity in the centre. Leaves on the peduncle 3–5, in a whorl; the lower leaf is the basal origin, proportional to or much longer than the inflorescence, narrowly lanceolate, up to 5 mm wide, gradually long-pointed, scaphoid on the cross section. Inflorescence 2–3-flowered; umbellate, pedicels of different lengths (0.5–45 mm). The perianths are different: external lanceolate, 15–18 mm long, 2–3 mm wide, pointed at the top, internal linear, shorter and narrower than the outer ones, rounded at the top, milky-white inside, bright green outside, white-edged. Anthers yellow, linear (3 mm long), opened - oblong (about 2 mm long). The capsule is ovate-rounded, less than half the length of the leaves. Seeds are flat.

**Figure 4.**
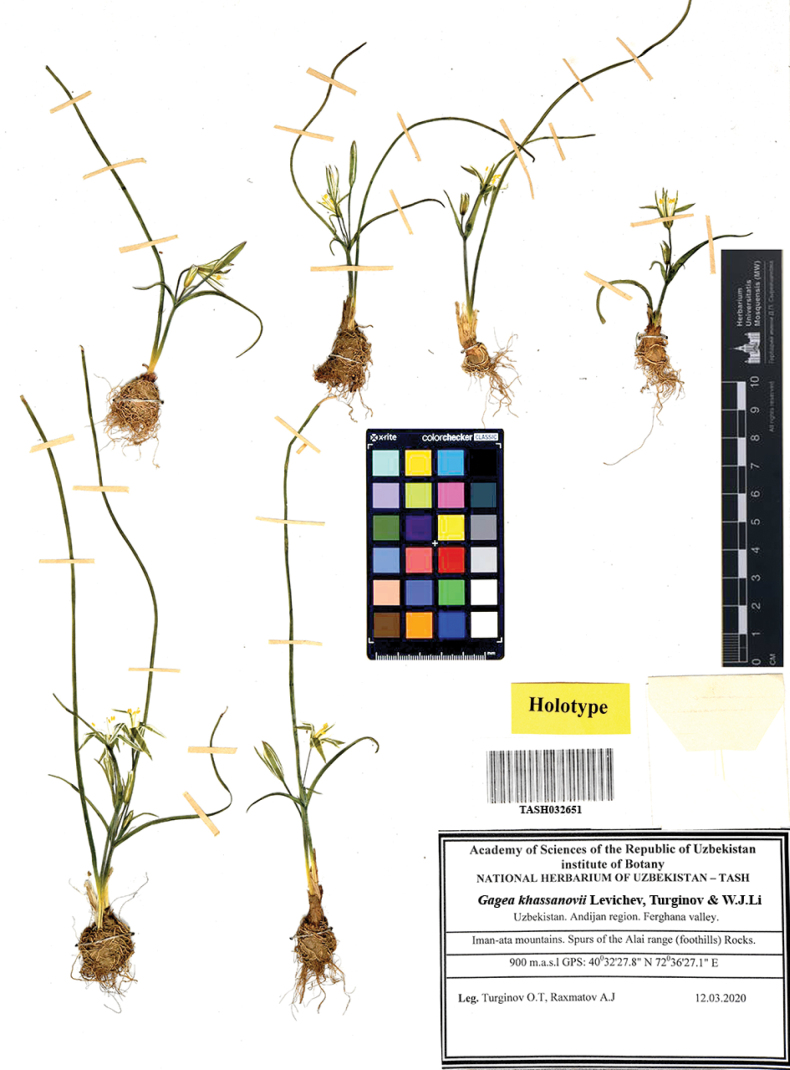
Typical herbarium specimen *Gageakhassanovii* Levichev, Turginov & W.J.Li, sp. nov.

##### Distribution and habitat.

**Figure 5.**
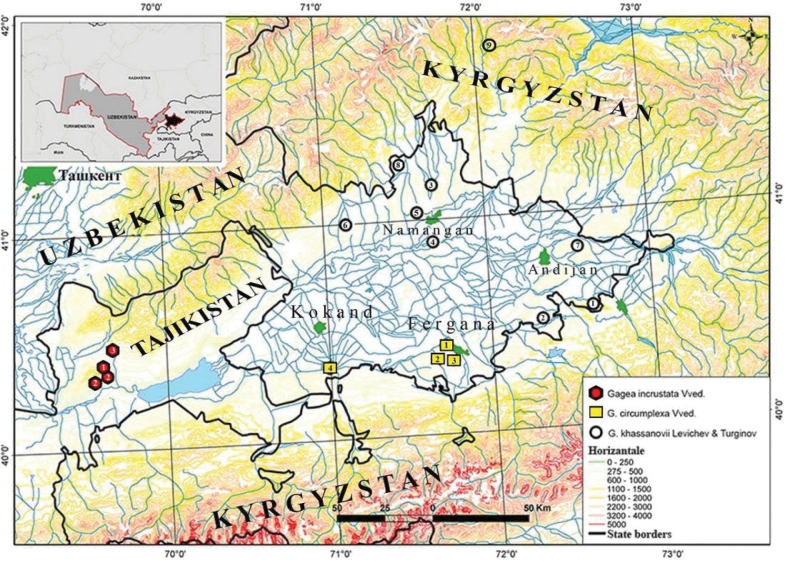
Schematic map of the Fergana Valley and habitat *G.incrustata*, *G.circumplexa* and *G.khassanovii* Levichev, Turginov & W.J.Li, sp. nov.

*Gageakhassanovii* is endemic to the eastern part of the Fergana Valley, occurring in the middle belt of the Chatkal and Alay Mountain ranges (Fig. 5). The species inhabits gravelly soils and rocky outcrops of mountain spurs, typically within well-drained, xerophytic habitats.

##### Phenology.

Flowering from March and fruiting in April.

##### Etymology.

The species is named in honour of the researcher and expert on the flora of Central Asia, Professor F. O. Khassanov.

##### Conservation status.

*G.khassanovii* is currently known only from the foothills of the Chatkal and Alay ranges in the Fergana Valley (Uzbekistan and Kyrgyzstan). Due to limited population data and the absence of repeated field observations (i.e. known only from the type locality), its conservation status remains uncertain. Based on the IUCN Red List Categories and Criteria (IUCN 2019), the species may be assessed as Data Deficient (DD). However, given its narrow distribution and potential threats to its habitat, it could plausibly fall under either Critically Endangered (CR) criteria B and C (e.g. Wagensommer et al. 2017; Wagensommer and Venanzoni 2021) or Near Threatened (NT) (Perrino et al. 2018), pending further field investigations.

##### Additional specimens (paratypes).

Uzbekistan • Andijan Region, Khojaabad Region, east-south-eastern part of the Fergana Valley, Kyrtashtau Mountains, near the Village of Imam-ata, 40°32'30.94"N, 72°36'29.88"E, 870 m. a.s.l. clay hillside. 12 III 2020. *D. A. Krivenko, O. A. Chernysheva, T. Kh. Makhkamov.* (IRK: IRK58734!). - Uzbekistan • Andijan Region, Jalakuduk Region, Fergana Valley, 1 km west of the village. South Alamyshik, high. 770 m a.s.l. clay hillside. 12 III 2020. 40°45'34.21"N, 72°35'25.36"E. *D. A. Krivenko, O. A. Chernysheva.* (IRK: IRK58735!).

##### Notes.

The Incrustatae section stands out for its taxonomically distinct position amongst other subdivisions of the genus, original morphological features and compact distribution in the western part of the Irano-Turan Region, including the Balkhash Region. At the moment, the section includes nine species and one more, the tenth taxon from the Peter the Great Ridge in the Pamir-Alay, is scheduled for description. All species are rare, few in number, with narrow local distribution in the foothills or in the mountains (Ajani et al. 2010)^1^.

Local rarity (Fig. 1) is one of the reasons for the superficial approach to the taxonomy of the representatives of the section, when several (up to 3–5) taxa from different sections and from remote geographic regions are combined under one name. Flat seeds, whorled inflorescence and bulb braid with thick roots, which are well preserved during herbarisation, become the reason for combining taxa from the sections *Graminifoliae* Levichev, *Platyspermum* Boiss., *Stipitatae* Davlianidze and *Incrustatae* (e.g. Wendelbo and Rechinger 1990; Zarrei et al. 2007: 572, etc.). Such associations are invalid since, in the section Graminifoliae, the root leaves are flat, as clearly (Wendelbo l. C.). In the *Platyspermum* section, the leaves are of a pentahedral type, narrow, grooved from above, and reinforced along the ribs with surface stiffening strands made of sclerenchyma. In the *Stipitatae* section, the leaves are round, unifacial and without sclerenchyma, often with a central fistu la, as in the *Incrustatae* section, but the inflorescence is necessarily branched (sometimes short-branched).

In Flora Iranica for *G.circumplexa*, a vast natural habitat is specified: from Libya to China (Wendelbo l. C.: 39), but the study of synonyms in the nomenclature quotation and studied samples from amongst those cited confirmed the inconsistency of such definitions and generalisations. In fact, in such a vast area under this name, all taxa with sclerified roots are listed in succession.

It should be emphasised that braiding with ageotropic, rigid roots is a very common feature characteristic of many species of all sections of the genus and even of vegetative bulbs.

This feature is a consequence of the two-cycle development of each shoot and the formation of different types of annual roots in successive cycles (Levichev 2001). Geotropically orientated, soft, thin roots appear in the autumn-spring period, feeding the aerial shoot. Much later, thick, rigid, ageotropically braiding and sclerified roots that rise to the bulb neck emerge from the bottom, which are produced by the bulb growing inside (Levichev 1999a). In the next year, this bulb develops an aerial shoot and soft (feeding) roots that literally pierce through the consumable storage scales of the previous year’s cycle (Levichev 1999b, Taf. 1).

Even in the first year of seedling life, this feature is observed: the primary (embryonic) root is feeding and the adventitious ageotropic rigid roots that appear later develop the first replacement bulb inside the cotyledon (Levichev 1999b, Taf. 3).

The first to draw attention to the thickened roots was Pascher (1942), calling them both “basket roots” (Korbchenwurzel) and “root basket” (Wurzelkorbchen) with the function of retaining moisture around the bulb. It should be added that the geotropic orientation of these roots to the root collar makes it possible to collect drops of scanty precipitation and dew in arid regions, flowing down to the shoot base and feeding the replacement bulb during the period of intensive increase in its mass. In addition, after dying off and over the course of many years, empty, large cells with thickened walls probably also perform the function of a soil velamen in a loose substrate, condensing vapours of air and retaining any minimum moisture content.

## Supplementary Material

XML Treatment for Gagea khassanovii

## Footnotes

Antithesis 5 of the identification key of the Incrustatae section (Ajani, l.c.: 70) contains a misprint in the form of an incorrect definition of “ G. setifolia sensu Wendelbo et Rech.f. 1990, p. p.”. G. setifolia Baker is a typical representative of the Platyspermum section (Ali and Levichev 2007, fig. 16 E–H) and G. aitchisoniana A. Terracc. Is from the Incrustatae section. The last taxon is the presence of two recognised names from different sections and makes the antithesis meaningless.

## ﻿Additional information

### Use of AI

No use of AI was reported.

### Author ORCIDs

Orzimat Turdimatovich Turginov https://orcid.org/0000-0001-7120-1895

Igor Germanovich Levichev https://orcid.org/0000-0002-1407-6474

Gulsauir Tanirbergen qizi Kurbaniyazova https://orcid.org/0000-0003-0103-0298

Farhod Isomiddinovich Karimov https://orcid.org/0000-0001-6129-4346

Lei Yang https://orcid.org/0009-0003-8888-5893

Wen Jun Li https://orcid.org/0000-0002-2932-0783

### Data availability

All of the data that support the findings of this study are available in the main text or Supplementary Information.

## Supplementary materials

Supplementary material 1 Gen Bank accession numbers Data type docx https://binary.pensoft.net/file/1383371This dataset is made available under the Open Database License (http://opendatacommons.org/licenses/odbl/1.0/). The Open Database License (ODbL) is a license agreement intended to allow users to freely share, modify, and use this Dataset while maintaining this same freedom for others, provided that the original source and author(s) are credited.Orzimat Turdimatovich Turginov, Igor Germanovich Levichev, Gulsauir Tanirbergen qizi Kurbaniyazova, Farhod Isomiddinovich Karimov, Lei Yang, Wen Jun Li

Supplementary material 2 Accession numbers and literature sources for DNA sequences of *Gagea* and related taxa Data type docx https://binary.pensoft.net/file/1383372This dataset is made available under the Open Database License (http://opendatacommons.org/licenses/odbl/1.0/). The Open Database License (ODbL) is a license agreement intended to allow users to freely share, modify, and use this Dataset while maintaining this same freedom for others, provided that the original source and author(s) are credited.Orzimat Turdimatovich Turginov, Igor Germanovich Levichev, Gulsauir Tanirbergen qizi Kurbaniyazova, Farhod Isomiddinovich Karimov, Lei Yang, Wen Jun Li

Supplementary material 3 Supplementary figure Data type jpg https://binary.pensoft.net/file/1383373This dataset is made available under the Open Database License (http://opendatacommons.org/licenses/odbl/1.0/). The Open Database License (ODbL) is a license agreement intended to allow users to freely share, modify, and use this Dataset while maintaining this same freedom for others, provided that the original source and author(s) are credited.Orzimat Turdimatovich Turginov, Igor Germanovich Levichev, Gulsauir Tanirbergen qizi Kurbaniyazova, Farhod Isomiddinovich Karimov, Lei Yang, Wen Jun Li
