# Supplementary material for: ﻿A new white-flowered species of Gagea (Liliaceae) from the Fergana Valley, Uzbekistan and Kyrgyzstan
Source: PhytoKeys. 2025 Jul 28;260:139–52. doi: 10.3897/phytokeys.260.151373 (PMC12322680; doi:10.3897/phytokeys.260.151373)
Supplement: Supplementary material 1 — Gen Bank accession numbers [file phytokeys-260-139_article-151373__-s001.docx]

Table S1. Gen Bank accession numbers

| **Taxa** | **Voucher information** | **ITS** | ***matK*** | ***psbA-trnH*** | ***rpl16*** | ***trnL-trnF* region** |
| --- | --- | --- | --- | --- | --- | --- |
| ***Gagea khassanovii 1*** |  | **PQ351917** | **PQ351603** | **PQ351603** | **PQ351603** | **PQ351603** |
| ***G. khassanovii 2*** |  | **PQ351916** | **PQ351604** | **PQ351604** | **PQ351604** | **PQ351604** |
| *Amana erythronioides* | Chase 742 (K) | EU912095 | AY624472 | EU939293 | EU912020 | EU912326 |
| *Erythronium japonicum* | Chase 780 (K) | EU912083 | AF485323 | EU939295 |  | EU912332 |
| *Fritillaria persica* | Chase 3496 (K) | AY616736 | AY624451 |  | AY624399 | EU912327 |
| *G. afghanica 1* | Zarrei & Golzarian 35257 (TUH)(Kew 23156) | EU912021 | EU912097 | EU939221 | EU912171 | EU912247 |
| *G. afghanica 2* | Zarrei & Golzarian 35223 (K, TUH)(Kew 23282) | EU912022 | EU912098 | EU939222 | – | EU912248 |
| *G. afghanica 3* | Zarrei & Golzarian 35207 (K, TUH)(Kew 23283) | EU912023 | EU912099 | EU939223 | – | EU912249 |
| *G. afghanica 4* | Levichev 52 (LE) | AM087953 | – | AJ973160 | – | AJ890373 |
| *G. aipetriensis* | Levichev 15 (LE) | AM087955 | – | AM049259 | – | AJ970178 |
| *G. alexeenkoana 1* | TUH-E BOT.EXP. 35305 (TUH)(Kew 23142) | EU912024 | EU912100 | EU939224 | EU912172 | EU912250 |
| *G. alexeenkoana 2* | TUH-E BOT.EXP. 35306 (TUH)(Kew 23297) | EU912030 | EU912106 | EU939229 | EU912179 | EU912257 |
| *G. alexeenkoana 3* | Levichev 34 (LE) | AM180458 | – | AM161460 | – | AM110257 |
| *G. algeriensis* | Chase 748 (K)  (Kew 748) | EU912088 | AY624470 | EU939280 | EU912232 | EU912311 |
| *G. altaica* | Levichev 51 (LE) | AM162670 | – | AJ973159 | – | AJ890374 |
| *G. bergii* | Zarrei & Golzarian 35222 (TUH)(Kew 23141) | EU912026 | EU912102 | – | EU912174 | EU912252 |
| *G. bohemica 1* | Andy Jones s.n. (Kew 7952) | – | EU912103 | – | EU912175 | EU912253 |
| *G. bohemica 2* | Germany: Saxony-  Anhalt | – | – | – | – | AJ437197 |
| *G. bohemica 3* | Levichev 50 (LE) | AM162672 | – | AM085142 | – | AJ969117 |
| *G. bohemica subsp.*  *bohemica 1* | Czech Republic:  Moravia | AJ427549 | – | AJ416370 | – | AJ419161 |
| *G. bohemica subsp.*  *bohemica 2* | Germany: Saxony-  Anhalt | AJ427548 | – | – | – | AJ419160 |
| *G. bohemica subsp.*  *Saxatilis* | Germany: Saxony-  Anhalt | AJ427547 | – | AJ416371 | – | AJ419159 |
| *G. bulbifera 1* | TUH-E BOT.EXP. 35713 (TUH)(Kew 23140) | EU912027 | EU912104 | EU939226 | EU912176 | EU912254 |
| *G. bulbifera 2* | TUH-E BOT.EXP. 35709 (TUH)(Kew 23166) | EU912028 | EU912105 | EU939227 | EU912177 | EU912255 |
| *G. bulbifera 3* | Levichev 2 (LE) | AM162669 | – | AM049260 | – | AJ969119 |
| *G. caelestis* | Levichev 44 (LE) | AM180456 | – | AJ973165 | – | AJ969118 |
| *G. capillifolia* | Levichev 42 (LE) | AM087951 | – | AJ973171 | – | AJ970177 |
| *G. capusii* | Levichev 24 (LE) | – | – | AM085143 | – | AJ969123 |
| *G. caroli-kochii* | TUH-E BOT.EXP. 35715 (TUH)(Kew 23139) | EU912029 | EU912170 | EU939228 | EU912178 | EU912256 |
| *G. chanae* | Zarrei 867 (K)(Kew 23270) | EU912082 | EU912167 | EU939298 | EU912245 | – |
| *G. chlorantha 1* | Zarrei & Kamrani  35192 (TUH)(Kew 23138) | EU912031 | EU912107 | EU939230 | EU912180 | EU912258 |
| *G. chlorantha 2* | Zarrei & Kamrani  35195 (TUH)(Kew 23167) | EU912032 | EU912108 | EU939231 | EU912181 | EU912259 |
| *G. chlorantha 3* | Zarrei & Zarre 778  (K, TUH)(Kew 23268) | EU912033 | EU912109 | EU939232 | EU912182 | EU912260 |
| *G. chlorantha 4* | Zarrei 872 (K, TUH)(Kew 23269) | EU912034 | EU912110 | EU939233 | EU912183 | EU912261 |
| *G. chlorantha 5* | Hikmat Abbas Al-Ani  & Danail & Danail Aoraha 9354 (K)(Kew 23275) | EU912035 | EU912111 | – | – | EU912262 |
| *G. chomutovae 1* | Zarrei & Golzarian 35214 (TUH)(Kew 23146) | EU912036 | EU912112 | EU939234 | EU912184 | EU912263 |
| *G. chomutovae 2* | Zarrei 35814 (TUH)(Kew 23168) | EU912037 | EU912113 | EU939235 | EU912185 | EU912264 |
| *G. chomutovae 3* | Levichev 37 (LE) | AM087950 | – | AM049262 | – | AJ970176 |
| *G. circumplexa 1* | Carter 721 (K)(Kew 23274) | EU912038 | EU912114 | EU939236 | EU912186 | EU912265 |
| *G. circumplexa 2* | Levichev 30 (LE) | AM265529 | – | AJ973172 | – | AJ969122 |
| *G. commutata 1* | Zarrei 876 (K, LE, TUH)(Kew 23336) | EU912039 | EU912115 | EU939237 | EU912187 | EU912266 |
| *G. commutata 2* | Dafni s.n. (Kew 23410) | EU912096 | – | EU939296 | EU912243 | EU912329 |
| *G. confusa 1* | TUH-E BOT.EXP. 35712 (TUH)(Kew 23169) | EU912041 | EU912117 | EU939239 | EU912189 | EU912268 |
| *G. confusa 2* | Zarrei & Zarrei 35266 (TUH)(Kew 23137) | EU912040 | EU912116 | EU939238 | EU912188 | EU912267 |
| *G. confusa 3* | Levichev 13 (LE) | AM087949 | – | AJ973173 | – | AJ890369 |
| *G. dayana* | Davis 8235 (K)(Kew 23273) | EU912042 | – | – | EU912190 | EU912269 |
| *G. dschungarica 1* | Zarrei 35815 (TUH)(Kew 23143) | EU912043 | EU912118 | EU939240 | EU912191 | EU912270 |
| *G. dschungarica 2* | Zarrei 35290 (TUH)(Kew 23170) | EU912044 | EU912119 | EU939241 | EU912192 | EU912271 |
| *G. dschungarica 3* | Levichev 14 (LE) | AM087952 | – | AJ973164 | – | AJ970175 |
| *G. eleonorae* | Levichev 57 (LE) | AM287274 | – | AJ973163 | – | AJ970179 |
| *G. exilis* | Moussavi & Tehrani 29971 (IRAN)(Kew 23182) | – | – | EU939242 | EU912193 | EU912272 |
| *G. filiformis* | Levichev 12 (LE) | AM180457 | – | AM161459 | – | AM084904 |
| *G. foliosa* | Italy: Sardegna 34697 (Z) | AM162676 | – | AM049258 | – | AJ969124 |
| *G. fragifera 1* | Zarrei 35820 (TUH)(Kew 23144) | EU912045 | EU912120 | EU939243 | EU912194 | EU912273 |
| *G. fragifera 2* | TUH-E BOT.EXP. 35711 (TUH)(Kew 23171) | EU912046 | EU912121 | EU939244 | EU912195 | EU912274 |
| *G. fragifera 3* | TUH-E BOT.EXP. 35307 (TUH)(Kew 23294) | EU912086 | EU912153 | EU939278 | EU912230 | EU912309 |
| *G. fragifera 4* | Zarrei & Zarrei 35265 (K, TUH)(Kew 23296) | EU912047 | EU912122 | EU939245 | EU912196 | EU912275 |
| *G. fragifera 5* | Italy: 12692 (CLU) | AM287285 | – | AM282995 | – | AM283102 |
| *G. fragifera 6* | Switzerland: Canton Graubuenden 10726  (ZT) | – | – | AM238531 | – | AJ890375 |
| *G. fragifera 7* | Bulgaria: Pirin-mountains 070407 (HAL) | AM162677 | – | AJ973158 | – | AJ890368 |
| *G. fragifera 8* | Levichev 29b (LE) | AM180455 | – | AM238521 | – | AM161467 |
| *G. gageoides 1* | Zarrei & Kamrani  35274 (TUH)(Kew 23172) | EU912049 | EU912169 | EU939247 | EU912198 | EU912277 |
| *G. gageoides 2* | TUH-E BOT.EXP. 35714 (TUH)(Kew 23145) | EU912048 | EU912168 | EU939246 | EU912197 | EU912276 |
| *G. gageoides 3* | Levichev 41 (LE) | AM162673 | – | AM161462 | – | AM084905 |
| *G. glacialis* | Marais 1565 (K)(Kew 23279) | – | – | – | EU912199 | EU912278 |
| *G. graeca 1* | Davis 40591 (K)(Kew 23339) | EU912077 | EU912159 | EU939285 | EU912235 | EU912316 |
| *G. graeca 2* | Greece: Crete, Lassithi plateau 099962 (HAL) | AJ810089 | – | AM049263 | – | AJ810090 |
| *G. graeca 3* | Greece: Lakonia | AJ810088 | – | – | – | – |
| *G. granulosa 1* | Levichev 11b (LE) | AM287278 | – | AM238517 | – | AM180463 |
| *G. granulosa 2* | Levichev 11a (LE) | AM265533 | – | AM238518 | – | AM180462 |
| *G. heldreichii* | Levichev 8 (LE) | AM265534 | – | AM161464 | – | AM180467 |
| *G. helenae* | Levichev 22 (LE) | AM265531 | – | AM161461 | – | AJ969120 |
| *G. helicophylla* | Levichev 35a (LE) | – | – | AM085145 | – | AM084901 |
| *G. hiensis* | Mongolia: Bogd-Ul Mountains 070426 (HAL) | AM287279 | – | AJ973169 | – | AJ890367 |
| *G. infrakamensis* | Levichev 10 (LE) | AM180459 | – | AM238519 | – | AM180471 |
| *G. iranica 1* | Zarrei & Golzarian 35210 (TUH)(Kew 23147) | EU912050 | EU912123 | EU939248 | EU912200 | EU912279 |
| *G. iranica 2* | Zarrei & Golzarian 35251 (TUH)(Kew 23173) | EU912051 | EU912124 | EU939249 | EU912201 | EU912280 |
| *G. lactea* | Levichev 53 (LE) | AM180452 | – | AJ973166 | – | AJ969125 |
| *G. libanotica* | Townsend 74/38 (K)(Kew 23338) | – | EU912160 | EU939286 | EU912236 | EU912317 |
| *G. lojaconoi* | Italy 9256 (CLU) | AM287272 | – | AM282997 | – | AM283106 |
| *G. lutea 1* | Zarrei 35285 (TUH)(Kew 23148) | EU912052 | EU912125 | EU939250 | EU912202 | EU912281 |
| *G. lutea 2* | Germany: Saxony-Anhalt, Rothenschirmbach | AJ488569 | – | AJ416368 | – | AJ488279 |
| *G. lutea 3* | Levichev 16 (LE) | AM265530 | – | AM161456 | – | AM110255 |
| *G. luteoides* | Baytor, T. ISTE 44270 (K)(Kew 23280) | EU912053 | EU912126 | EU939251 | EU912203 | EU912282 |
| *G. megapolitana* | Henker (HAL) | – | – | AM161455 | – | AM084902 |
| *G. minima* | Germany: Saxony-  Anhalt | AJ427546 | – | AJ416374 | – | AJ419164 |
| *G. nakaiana* | Levichev 17 (LE) | AM180454 | – | AM161457 | – | AM110256 |
| *G. olgae* | Levichev 3 (LE) | – | – | AM085144 | – | AM161465 |
| *G. ova 1* | Levichev 39b (LE) | AM287277 | – | AM265588 | – | AM180466 |
| *G. ova 2* | Levichev 39a (LE) | AM287276 | – | AM238526 | – | AM180465 |
| *G. pauciflora* | Mongolia: Ulan Bator 070423 (HAL) | AM409330 | – | AJ973168 | – | AJ890372 |
| *G. peduncularis* | Davis 40349 (K)(Kew 23333) | EU912054 | EU912127 | EU939252 | EU912204 | EU912283 |
| *G. podolica* | Levichev 21 (LE) | AM409334 | – | AM238525 | – | AM084903 |
| *G. pomeranica 1* | Germany: Mecklenburg-Western Pomeranica 095846 (HAL) | AJ429193 | – | AJ429194 | – | – |
| *G. pomeranica 2* | Germany: Saxony-  Anhalt 095842 (HAL) | AJ427543 | – | AJ416375 | – | AJ419167 |
| *G. pratensis 1* | Germany: Saxony-  Anhalt | AJ437203 | – | AJ416372 | – | AJ437196 |
| *G. pratensis 2* | Germany: Brandenburg | AJ437202 | – | – | – | AJ437195 |
| *G. pratensis 3* | Germany: Saxony-  Anhalt | AJ437201 | – | – | – | AJ419162 |
| *G. pusilla* | Levichev 18 (LE) | – | – | AM161458 | – | AM180464 |
| *G. reticulata 1* | Zarrei & Golzarian 35260 (TUH)(Kew 23150) | EU912056 | EU912129 | EU939254 | EU912206 | EU912285 |
| *G. reticulata 2* | Zarrei & Kamrani  35196 (TUH)(Kew 23174) | EU912058 | EU912131 | EU939256 | EU912208 | EU912287 |
| *G. reticulata 3* | Zarrei & Kamrani  35186 (K, TUH)(Kew 23284) | EU912060 | EU912133 | EU939258 | EU912210 | EU912289 |
| *G. reticulata 4* | Zarrei & Ajani 832 (IRAN, K, M, TUH)(Kew 23287) | EU912061 | EU912134 | EU939259 | EU912211 | EU912290 |
| *G. reticulata 5* | Levichev 56 (LE) | – | – | AJ973162 | – | – |
| *G. setifolia 1* | Zarrei 35289 (TUH)(Kew 23149) | EU912055 | EU912128 | EU939253 | EU912205 | EU912284 |
| *G. setifolia 2* | Zarrei & Golzarian 35246 (TUH)(Kew 23151) | EU912063 | EU912136 | EU939261 | EU912213 | EU912292 |
| *G. setifolia 3* | Zarrei & Golzarian 35254 (TUH)(Kew 23175) | EU912064 | EU912137 | EU939262 | EU912214 | EU912293 |
| *G. setifolia 4* | Heydari 30547 (K)  (Kew 23267) | EU912065 | EU912138 | EU939263 | EU912215 | EU912294 |
| *G. setifolia 5* | Zarrei 1017 (K)(Kew 23272) | EU912066 | EU912139 | EU939264 | EU912216 | EU912295 |
| *G. setifolia 6* | Mohammadi 35198 (K, TUH)(Kew 23285) | EU912067 | EU912140 | EU939265 | EU912217 | EU912296 |
| *G. setifolia 7* | Zarrei & Zarrei 35268 (K, TUH)(Kew 23290) | EU912068 | EU912141 | EU939266 | EU912218 | EU912297 |
| *G. setifolia 8* | Zarrei & Golzarian 35213 (K, TUH)(Kew 23291) | EU912069 | EU912142 | EU939267 | EU912219 | EU912298 |
| *G. soleirolii* | Montserrat *et al.* s.n. (Kew 20651) | – | EU912166 | EU939297 | EU912244 | EU912330 |
| *G. spathacea 1* | Levichev 37 (LE) | – |  | AJ973174 |  | AJ969126 |
| *G. spathacea 2* | Germany: Saxony-  Anhalt 095844 (Hal) | AJ427541 | – | AJ416369 | – | AJ419166 |
| *G. stipitata 1* | Zarre & Zarrei 35297 (TUH)(Kew 23154) | EU912070 | EU912143 | EU939268 | EU912220 | EU912299 |
| *G. stipitata 2* | Zarrei & Kamrani 35275 (TUH)(Kew 23176) | EU912071 | EU912144 | EU939269 | EU912221 | EU912300 |
| *G. stipitata 3* | Zarrei & Kamrani 35197 (TUH)(Kew 23177) | EU912072 | EU912145 | EU939270 | EU912222 | EU912301 |
| *G. stipitata 4* | Zarrei & Golzarian 35215 (K, TUH)(Kew 23293) | EU912073 | EU912146 | EU939271 | EU912223 | EU912302 |
| *G. tenera 1* | Zarrei & Golzarian 35256 (TUH)(Kew 23152) | EU912074 | EU912147 | EU939272 | EU912224 | EU912303 |
| *G. tenera 2* | Zarrei & Golzarian 35219 (K, TUH)(Kew 23281) | EU912075 | EU912148 | EU939273 | EU912225 | EU912304 |
| *G. transversalis* | Levichev 56 (LE) | AM162671 | – | AJ973167 | – | AJ890370 |
| *G. triflora 1* | Furse & Miyoshi 26159 (K)(Kew 23409) | – | – | – | EU912246 | EU912331 |
| *G. triflora 2* | Levichev 46 (LE) | AM162674 | – | AM049261 | – | AJ890377 |
| *G. uliginosa 1* | TUH-E BOT.EXP. 35304 (TUH)(Kew 23153) | EU912089 | EU912155 | EU939281 | EU912233 | EU912312 |
| *G. uliginosa 2* | Moussavi *et al.* 30018 (IRAN)(Kew 23288) | EU912090 | EU912157 | EU939283 | – | EU912314 |
| *G. uliginosa 3* | Rawi & Serhang 18286 (K)(Kew 23277) | – | EU912156 | EU939282 | EU912234 | EU912313 |
| *G. vegeta 1* | Shafii 475 (Shahed University Herbarium)(Kew 23181) | EU912076 | EU912149 | EU939274 | EU912226 | EU912305 |
| *G. vegeta 2* | Zarrei and Zarre, 1033 (K, TUH)(Kew 23335) | – | EU912150 | EU939275 | EU912227 | EU912306 |
| *G. vegeta 3* | Levichev 32 (LE) | AM287275 | – | AM238520 | – | AM180468 |
| *G. villosa 1* | Zarrei & Kamrani 35273 (TUH)(Kew 23178) | EU912084 | EU912151 | EU939276 | EU912228 | EU912307 |
| *G. villosa 2* | Zarrei & Golzarian 35247 (TUH)(Kew 23179) | EU912085 | EU912152 | EU939277 | EU912229 | EU912308 |
| *G. villosa 3* | Zarrei & Golzarian 35253 (TUH)(Kew 23295) | EU912087 | EU912154 | EU939279 | EU912231 | EU912310 |
| *G. villosa 4* | Levichev 7 (LE) | AM180453 | – | AJ973170 | – | AM238538 |
| *G. villosa 5* | Germany: Saxony-  Anhalt | AJ427545 | – | AJ416373 | – | AJ419163 |
| *G. wendelboi* | Matin 35605 (IRAN)(Kew 23183) | EU912091 | EU912158 | EU939284 | – | EU912315 |
| *G. cf. reticulata* | Zarrei & Zarre 1032 (K)(Kew 23271) | EU912059 | EU912132 | EU939257 | EU912209 | EU912288 |
| *G. cf. setifolia 1* | Zarre 1009 (K, TUH)(Kew 23286) | EU912025 | EU912101 | EU939225 | EU912173 | EU912251 |
| *G. cf. setifolia 2* | Zarrei & Golzarian 35252 (K, TUH)(Kew 23292) | EU912062 | EU912135 | EU939260 | EU912212 | EU912291 |
| *G. sp.* | Zarrei & Kamrani 35194 (TUH)(Kew 23155) | EU912057 | EU912130 | EU939255 | EU912207 | EU912286 |
| *Lilium ledebourii* | Zarrei s.n. (TUH)(Kew 23346) | – | EU912165 | EU939299 | EU912242 | EU912328 |
| *Lloydia delicatula* | AGSES 212 (K)(Kew 23340) | EU912079 | – | – | – | EU912320 |
| *L. flavonutans* | AGSES 77 (K)(Kew 23341) | EU912080 | – | – | EU912238 | EU912321 |
| *L. oxycarpa* | ACE 137 (K)(Kew 23342) | EU912081 | – | EU939289 | – | EU912322 |
| *L. serotina 1* | Jones s.n. (K)(Kew  1004) | EU912092 | AY624471 | EU939288 | – | EU912319 |
| *L. serotina 2* | Levichev 45a (LE) | AM087956 | – | AM238530 | – | AJ890376 |
| *L. serotina 3* | Bulgaria: Ovtscharez 074806 (HAL) | – | – | AJ585048 | – | AJ585049 |
| *L. yunnanensis* | Luo, Yi-bo 64 (K)(Kew 23337) | EU912078 | EU912161 | EU939287 | EU912237 | EU912318 |
| *Tulipa clusiana* | Zarrei 35183 (TUH)(Kew 23348) | EU912093 | EU912162 | EU939290 | EU912239 | EU912323 |
| *T. lehmanniana* | Zarrei & Golzarian 35228A (TUH)(Kew 23349) | EU912094 | EU912163 | EU939291 | EU912240 | EU912324 |
| *T. uniflora* | Chase 751 (K) | – | EU912164 | EU939292 | EU912241 | EU912325 |
