# Supplementary figures and images for: ﻿A new white-flowered species of Gagea (Liliaceae) from the Fergana Valley, Uzbekistan and Kyrgyzstan
Source: PhytoKeys. 2025 Jul 28;260:139–52. doi: 10.3897/phytokeys.260.151373 (PMC12322680; doi:10.3897/phytokeys.260.151373)

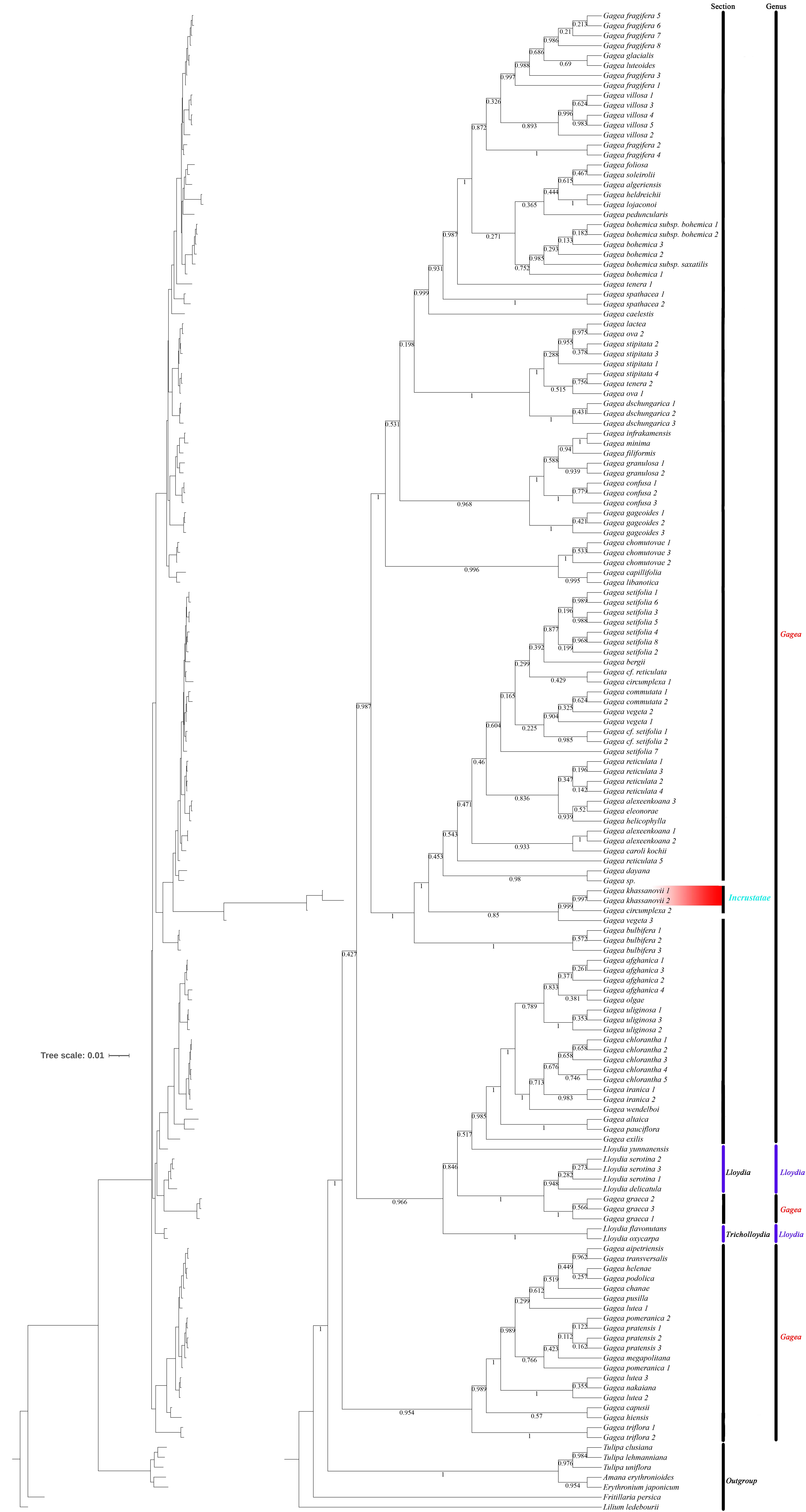

Supplement: Supplementary material 3 — Supplementary figure [file phytokeys-260-139_article-151373__-s003.jpg]
